# Sperm Cells Induce Distinct Cytokine Response in Peripheral Mononuclear Cells from Infertile Women with Serum Anti-Sperm Antibodies

**DOI:** 10.1371/journal.pone.0044172

**Published:** 2012-08-31

**Authors:** Miloslav Kverka, Zdenka Ulcova-Gallova, Jirina Bartova, Jan Cibulka, Katarina Bibkova, Zdenka Micanova, Helena Tlaskalova-Hogenova

**Affiliations:** 1 Institute of Microbiology, Academy of Sciences of the Czech Republic, Prague, Czech Republic; 2 Department of Gynecology and Obstetrics, Charles University and Faculty Hospital, Pilsen, Czech Republic; 3 Institute of Dental Research, General University Hospital, First Faculty of Medicine of the Charles University, Prague, Czech Republic; Institut Jacques Monod, France

## Abstract

**Background and Aims:**

Anti-sperm antibodies in can markedly reduce the likelihood of natural conception. The etiology of this anti-sperm immunity in human females is unknown. We compared the cytokine response of peripheral blood mononuclear cells (PBMCs) from infertile patients with or without anti-sperm antibodies (ASA) and fertile women.

**Methodology/Principal Findings:**

We cultivated the PBMCs together with sperm antigens (whole cells or cell lysate), and screened the supernatants for 40 cytokines by antibody array. When stimulated with whole sperm cells, the PBMCs from patients with ASA produce less IL-3, IL-11, IL-13, ICAM-1, GCSF and more IL-2, IL-4 and IL-12p70 as compared to healthy women. PBMCs from patients with ASA produce typically less IL-13, IL-7, IL-17 and MIG, and more MIP-1β and IL-8, as compared to PBMCs from patients without ASA. In response to sperm cell lysate, PBMCs from infertile women without ASA respond initially by increase in production of growth factors (GCSF, GM-CSF and PDGF-BB) followed by increase in chemokines (e.g. IL-8, MCP-1 and MIP-1β).

**Conclusions:**

Cellular immune responses to sperm antigens, measured by production of cytokines, differ among infertile women with ASA, infertile women without ASA and healthy women. This difference could play an important role in the initial steps of the infertility pathogenesis.

## Introduction

Presence of anti-sperm antibodies (ASA) in serum of infertile women suggests ongoing immune responses to sperm antigens. However, the studies describing cellular immune responses to sperm antigens in these patients are scarce. Cytokines and chemokines are one of the most important means of communication and effector function of immune cells leading to efficient protection of the host, but they can also drive the pathology of several immune mediated diseases. Cytokines act mainly in a paracrine and autocrine manner so they are released and consumed locally at the site where immune reaction occurs, driving the development of effector lymphocytes. These lymphocytes can leave the microenvironment and travel through the circulation to another sites of the body and even to another individual through breast-feeding, thus spreading the locally-induced immune response [1], [2].

Cytokines produced during lymphocyte-sperm interaction could cause the infertility by both influencing the cellular immune response to sperm, and also by impairing fertilization process and early embryo development, as demonstrated in animal models [3], [4]. The process of fertilization and implantation involves the interaction of the human sperm and oocyte, shortly afterwards blastocyst and the uterine epithelium. Cytokines are able to influence, both sperm – oocyte fusion, and early embryo development, thus ballancing between physiological embryo development and embryo resorption/or missed or spontaneus miscarriage [5], [6]. Moreover, several autoimmune factors have been implicated to influence implantation processes. A significant part of pregnancy losses is associated with various etiologies, including autoimmune, iso-immune, and cellular immune abnormalities [7], [8].

Our aim was to compare the cellular reactivity of peripheral blood mononuclear cells (PBMCs) of infertile women, to sperm cells or sperm cell lysate, with those of fertile women and teenage virgins (virgo intacta). For this purpose, we used protein array, capable to detect wide spectrum of cytokines, chemokines and growth factors.

## Results

**Figure 1:**
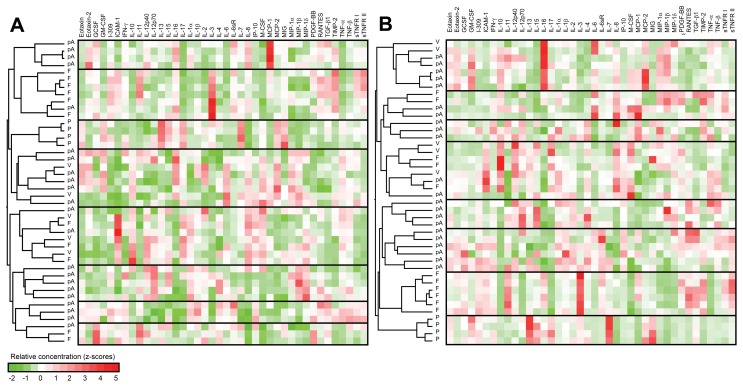
Heat map of cytokine levels. PBMCs from patients with and without ASA produce distinct cytokine patterns after their 3- (A) and 5- (B) day cultivation with whole sperm antigen. The samples were clustered based on the similarity of their cytokine profiles into 8 clusters, using eight-way unsupervised clustering algorithm with a top down repeated bisection approach using the correlation coefficient to calculate the similarity between the protein productions. F = fertile, V = teenage virgins, P = patients without ASA, pA = patients with ASA.

To compare the cytokine profiles, we arranged the samples by the similarity in abundance of the 40 cytokines produced by the PBMCs using an unsupervised clustering algorithm (Figure 1). This analysis revealed that while patients and fertile women segregated into clusters, teenage virgins do not have typical cytokine profile. The clustering cannot be explained either by age, or by previous IVF, or by history of spontaneous or artificial abortion.

To identify the significant differences in cytokine production between patients with ASA and fertile women after the incubation of their PBMCs with whole sperm cells, we performed SAM analyses. By these analyses, we have identified 5 or 7 differentially produced cytokines (FDR cut-off of 10%) for 3 or 5 days of incubation respectively (Table 1). Next, by employing similar approach, we compared the cytokine production of PBMCs from patients with ASA to that of PBMCs from patients without ASA after their cultivation with sperm cells. We identified 11 differently produced cytokines for both 3 and 5 days of incubation respectively (Table 2). There is a decrease in ICAM-1 and IL-3 and increase in eotaxin and TNF-α as compared to fertile women (Table 3) in all patients (with and without ASA).

**Table 1:** Differentially produced proteins by PBMCs from patients with ASA as compared to fertile women, after their 3- or 5-day incubation with whole sperm cells.

Cultivation	Protein	FDR (q)	Fold change
	IL-3	q<0.05	0.42
	IL-13	q<0.05	0.47
**3 days**	ICAM-1	q<0.05	0.63
	IL-11	q<0.05	0.63
	GCSF	q<0.05	0.77
	IL-3	q<0.05	0.66
	TIMP-2	q<0.05	0.88
	ICAM-1	q<0.05	0.91
**5 days**	PDGF-BB	q<0.05	0.97
	IL-12p70	q = 0.10	2.04
	IL-4	q = 0.10	3.38
	IL-2	q = 0.10	4.52

Fold change less than 1 indicates lower and more than 1 indicates higher abundance in patients with ASA as compared to fertile women. FDR, false discovery rate. Sample size: patients with ASA (n = 22); fertile women (n = 11).

**Table 2:** Differentially produced proteins by PBMCs from patients with ASA as compared to patients without ASA, after their 3- or 5-day incubation with whole sperm cells.

Cultivation	protein	FDR (q)	Fold change
	IL-13	q<0.05	0.03
	IL-7	q<0.05	0.03
	MIG	q<0.05	0.21
	IL-17	q<0.05	0.32
	MCP-2	q<0.05	0.40
**3 days**	IL-15	q<0.05	0.46
	GM-CSF	q<0.05	0.47
	IL-11	q<0.05	3.63
	Eotaxin-2	q<0.05	10.04
	MIP-1β	q<0.05	26.88
	IL-8	q<0.05	27.35
	IL-13	q<0.05	0.04
	IL-7	q<0.05	0.05
	MIG	q<0.05	0.31
	GM-CSF	q<0.05	0.41
	IL-15	q<0.05	0.48
**5 days**	IL-17	q<0.05	0.49
	IL-3	q<0.05	0.64
	MCP-2	q<0.05	0.67
	I-309	q<0.05	0.80
	Eotaxin	q<0.05	0.86
	MIP-1β	q<0.05	23.69

Fold change less than 1 indicates lower abundance and more than 1 indicates higher abundance in patients with ASA as compared to patients without ASA. Only significant (q<0.05) proteins are shown. FDR, false discovery rate. Sample size: patients with ASA (n = 22) and patients without ASA (n = 4).

**Table 3:** Differentially produced proteins by PBMCs from all infertile patients as compared to fertile women, after their 3- or 5-day incubation with whole sperm cells.

Cultivation	protein	FDR (q)	Fold change
	ICAM-1	q<0.05	0.57
3 days	IL-3	q<0.05	0.48
	Eotaxin	q<0.05	3.22
	TNF-α	q<0.05	2.78
	TIMP-2	q<0.05	0.77
	ICAM-1	q<0.05	0.85
	PDGF-BB	q<0.05	0.88
	IL-3	q<0.05	0.72
5 days	IL-2	q<0.10	4.28
	IL-12p70	q<0.10	1.94
	Eotaxin	q<0.10	5.42
	IL-4	q<0.10	3.09
	MCP-2	q<0.10	4.16
	GM-CSF	q<0.10	5.92

Fold change less than 1 indicates lower and more than 1 indicates higher abundance in patients with ASA as compared to fertile women. Sample size: infertile patients (n = 26); fertile women (n = 11).

To analyze the changes in cytokine production over time we compared the production of cytokines by PBMCs from patients, fertile women or teenage virgins after these cells were cultivated with whole sperm cells for 3 or 5 days respectively. The cytokine profiles of untreated cells do not change over time except for slight increase of GM-CSF in patients without ASA and IL-8 in patients with ASA. The production of cytokines by PBMCs stimulated by whole sperm cells is generally higher, but the pattern differs significantly between individual subjects. While the PBMCs from teenage virgins do not change the cytokine production, there is a significant decrease in IL-7 production in PBMCs from patients without ASA and increase in IL-6 in patients with ASA and a significant increase in IL-6 and IL-8 in PBMCs from fertile women between 3 and 5 days of incubation. PBMCs from patients have slightly different dynamics of cytokine production than PBMC from fertile women.

**Figure 2:**
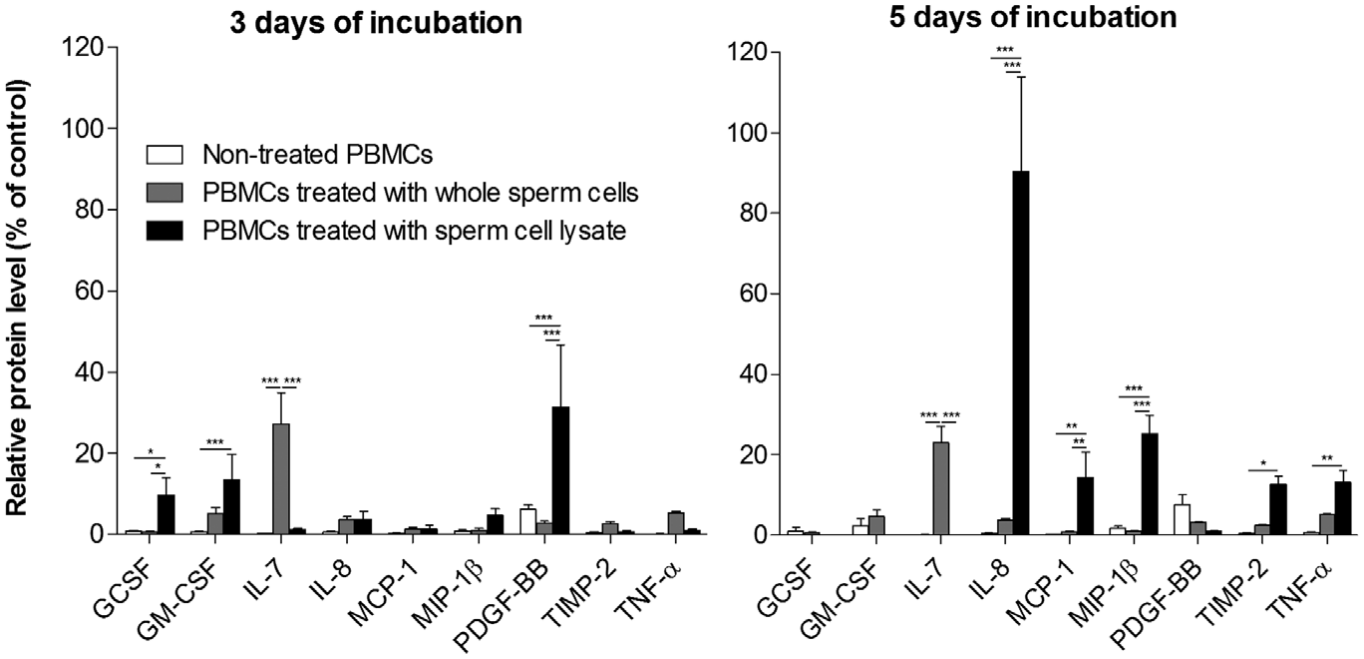
The changes in cytokine production elicited by whole sperm cells or by their lysate. PBMCs from each infertile patient without serum ASA (n = 4) was incubated either with whole sperm cells or by sperm cell lysate, for either 3 or 5 days. The differences are significant as calculated by pair-matched ANOVA with Bonferroni multiple comparison test (*p<0.05, ** p<0.01, *** p<0.001). Only those cytokines, significantly different in at least one time-point are included.

Next, we used similar approach to analyse the changes in the cytokine production after the PBMCs from the same female patient were treated with either whole sperm cells or sperm cell lysate. Treatment with whole sperm cells typically increases IL-7 in both 3-day and 5-day culture. Sperm cell lysate initially led to increase in growth factors, which are later replaced by increase in chemokines. The significant differences in cytokine production after 3 or 5 days of incubation are shown on Figure 2.

## Discussion

The presence of high titers of ASA in serum, seminal plasma, or in ovulatory cervical mucus is associated with fertilization failure [9], [10], [11]. ASA can cause infertility by binding to the surface of spermatozoa, thereby blocking their penetrance through cervical mucus and interfering with sperm-egg interaction. Antigen-specific immune response to sperm antigens can be initiated by soluble sperm antigens during penetration through cervical area, uterine and tubal mucous membrane [11], [12]. As compared to antigen-specific humoral immune response, the specific cellular immune responses to sperm cells or sperm antigens in humans is only rarely investigated [13]. The interaction between T, B, and natural killer cells determinine if the embryo is accepted or rejected [5], [6], [14]. The result of immune response to the sperm antigens is greatly influenced by cytokine environment at the time when immunocompetent cell encounters the antigen. Besides this crucial immuno-modulatory function, cytokines are very important in implantation and embryo development [14]. The disturbance in cytokine environment could, therefore, play a significant role in mechanisms of the immune-mediated infertility either by disturbing the balance of immune system regulation or by directly interfering with fertilization process and early embryo development [15]. Moreover, the cytokine network operating in the female reproductive tract is under strong influence of the ovarian steroid hormones, so the hormonal state during the priming could strongly influence the immune response [16]. Understanding the cytokine function during reproduction is also complicated by the pleiotropy and redundancy of cytokine action and by the fact that overall cytokine environment shapes the effects of individual cytokines [17]. This complexity of immune mechanisms involved in fertilization causes difficulty when link of one particular cytokine to the pathology is desirable. Use of multiplex methods, capable to analyze many cytokines at once, may bring more relevant data regarding the biological effect than analysis of single cytokine, yet still it cannot entirely solve the above mentioned problem. By comparing the cellular response to sperm antigens between infertile women with ASA and healthy women by antibody array, we can suggest which pathogenetic mechanisms are active in this type of infertility.

In this study, we found that sperm antigens influence the cytokine response in infertile women with ASA differently, as compared to fertile women, or to infertile women without ASA. These changes were in cytokines characteristic for distinct functional T cell subsets, including typical Th1-type (IL-2), Th2- or NK-T-type (IL-4 and IL-13), and Th17-type (IL-17) cytokines. Moreover, we found changes in pro-inflammatory cytokines and chemokines (e.g. eotaxins, MIPs, IL-8, IL-12p70, and TNF-α), and in growth factors (e.g. IL-7, GCSF, GM-CSF). These pronounced changes suggest quite extensive dysregulation of immune response to sperm in infertile women involving several types of cells, and point at the involvement of several pathogenetic mechanisms. These mechanisms include enhancement of the immune reactivity with slight shift towards Th2-type of response and lag of the embryo implantation and its growth.

The increase in IL-2 and IL-4 production by whole sperm cells-stimulated PBMCs of infertile patients with ASA, as compared to controls, could create the environment that lead to generation of Th2-type of response and resulting in antibody production. This change could be the basis of the main pathogenetic feature - ASA. PBMCs from infertile women with ASA produce less IL-11 early upon stimulation with sperm cells as compared to healthy women. IL-11 is a pleiotropic cytokine from the IL-6 family that stimulate hematopoiesis and important role in embryo implantation [14]. Its deficiency during pregnancy is associated with infertility and miscarriage during the first trimester of pregnancy [18].

Decreased production of ICAM-1 by PBMCs of infertile women with ASA as compared to healthy controls is of particular interest, suggesting more complex interplay of factors involved in diapedesis. ICAM-1 is a glycoprotein expressed on the surface of several cell types, including leukocytes and endothelial cells that binds to the lymphocyte function-associated antigen-1 (LFA-1), expressed on leukocytes, thus mediating leukocyte extravasation and interaction [19]. High levels of soluble (s) ICAM-1, a shedding form of ICAM-1, can disturb the adhesions that occur between immune cells and their targets and thereby prevent an immunological reaction [20]. Therefore, its low production by PBMCs from patients with ASA could result in higher ability of reactive leukocyte to exit the blood stream and initiate the immune response in tissues, resulting in shift towards inflammation. It is important to note, however, that high levels of sICAM-1 are often detected in serum of patients with inflammatory condition [21], [22], which could be explained by existence of an anti-inflammatory feedback on the level of whole organism.

PBMCs from our patients with ASA produce less IL-13 and more IL-12 after their cultivation with sperm cells, as compared to healthy controls. As a key inducer of Th1-type inflammatory responses, IL-12 is involved in autoimmune tissue destruction. IL-13, on the other hand, is described as an anti-inflammatory cytokine, which inhibits the production of inflammatory cytokines by LPS-activated macrophages [23]. This change in patients with ASA could result in deregulated inflammation with subsequent infertility. Similar changes were found in peritoneal fluid of infertile women with moderate or severe endometriosis, suggesting that these distinct causes of infertility could have certain mechanisms in common [24]. Moreover, decrease in IL-13 could be also lead to impairment in angiogenesis, since IL-13 was identified as an important pro-angiogenic factor acting mainly through a soluble VCAM-1/α4 integrin pathway [25].

There is a striking difference in cytokine response to sperm cell by PBMCs from infertile patients with ASA as compared to those from patients without ASA. When we compared cytokine spectra between both groups of infertile patients, we found that e.g. IL-13, IL-7, IL-17, and MIG are higher in patients without ASA, and IL-8 and MIP-1β are higher in patients with ASA. This suggests that these forms of infertility differ in antigen-specific immune response to sperm cells not only on humoral, but also on cellular level.

PBMCs from infertile patients without ASA produce significantly higher amounts of IL-7 after their cultivation of with whole sperm cells-antigen, as compared to either unstimulated or sperm cell lysate-stimulated PBMCs. This cytokine is multifunctional growth factor, known to strongly promote the proliferation of activated T cells [26]. The role of IL-7 in the physiological reproduction is not well documented; it is believed that due to its general growth promoting activities it stimulates folliculogenesis during *in vitro* fertilization [27]. Its high production by PBMCs from infertile patients without ASA could lead to more aggressive cellular response in these patients, which can compensate for the lack of above-mentioned pathogenetic role of ASA in these patients. We found the response to whole sperm cells is generally less diverse than response to sperm cell lysate. These antigens induce high production of IL-8, MCP-1, TNF-α and MIP-1β, but not IL-7, as compared to either non-stimulated or whole sperm-stimulated PBMCs, which correspond to protective immune response. These differences also point out the differences between the response to extracellular and to intracellular antigens.

There are several mechanisms involved in immune response to sperm cells in infertile women with ASA and the presence of ASA are only one of them. Here, we investigated, often-neglected role of cellular immune response in pathogenesis of this disorder, using exclusively human samples. We described that PBMCs from infertile women with ASA produce different cytokines when encounter the sperm cells, as compared to healthy controls. This type of cytokine response could contribute to pro-inflammatory environment, Th2 favorable conditions or impairment of angiogenesis leading to infertility. Some of these cytokines could influence the cell proliferation and recruitment or participate in the sperm cell-specific immune response. We showed that cellular response to sperm cells is different in infertility with and without ASA, suggesting that cellular response differs between these two types of infertility. Although the intricate connections of inter-cellular communication make the clear connection between one particular cytokine and infertility with ASA impossible, they clearly show the disturbance of the cellular immune response in infertile women with ASA, when exposed to sperm. The dysregulation of the immune response with shift towards Th2-type of response suggest how the initial disturbance in cellular immune response could result in ASA, yet more research is needed to prove this hypothesis and link it with particular type of the cell.

## Materials and Methods

### Patients

We investigated the reactivity of PBMCs from a total of 22 infertile women aged 31.5 (25–40) years (median (range)) with serum IgG sperm agglutinating antibodies (titer higher than 1∶64) proven by tray microagglutination test (TAT) and indirect mixed antiimunoglobulin reaction (i-MAR) tests [9], [28]. We also investigated the reactivity of PBMCs from 4 infertile women aged 29.5 (29–30) (median (range)) without ASA, defined as negative TAT. Women from both these groups attend Special Division for Infertility and Immunology of Reproduction at the Department of Obstetrics and Gynaecology, Charles University and Faculty Hospital, Pilsen, Czech Republic, and have normal gynecological, genetic and hormonal findings. We also studied reactivity of PBMC of 11 fertile healthy women aged 36.5 (24–43) years (median (range)), known to conceived and gave a birth spontaneously to two healthy children, have regular sexual intercourse and menstrual cycle, and do not use hormonal contraceptives, and five healthy teenage virgins aged 15.0 (13–17) years, (median range) without any contact with semen and with regular menstrual cycle. To minimalize the effect of steroid hormones on PBMCs function, we scheduled the sample collection to the time between the end of menstrual bleeding and the expected time of ovulation in each studied subject. The study was approved by the Local Research Ethics Committee of University Hospital in Pilsen, and written informed consent was obtained from all participants or from parents of teenage female virgins.

### Detection of Sperm Antibodies

In all subjects of this study, we performed TAT and i-MAR tests according previously published guidelines for examination of the serum ASA [9], [28]. TAT test serves as an initial screening examination. The blood from studied subjects was collected by venipuncture into evacuated tubes and centrifuged. Isolated serum was inactivated by heat (56°C for 30 min) and stored at −20°C until analysis. We performed the TAT test by adding 5 µL of inactivated, geometrically diluted, serum and 1 µL of motile donor sperm (40×10^6^/ml), isolated by “swim-up technique” into microchambers covered by paraffin oil. After the incubation (2 h at 37°C), the immunological reaction was evaluated under inverted microscope at 200×magnification. Agglutination of the sperm cells at dilution at least 1∶64 was considered as a positive result.

The i-MAR test was performed to analyze the anti-sperm response in IgG and IgA class. One microliter of native sperm suspension, 1 µL of inactivated serum, 1 µL of glutaraldehyde-fixed sheep erythrocytes pre-coated with human IgG, and IgA, were mixed together. Then, 1 µL of the corresponding antiserum anti-IgG, anti-IgA (Behringer, Hannover, Germany) was added. Finally, the mixture was covered with a cover slip and incubated in humid Petri chamber for 5–10 min. The result of the sperm agglutination reaction was watched under the inverted microscope at 200×magnification. The i-MAR test was considered as positive, if more than 49% of motile spermatozoa were involved in mixed agglutinates (spermatozoa and sheep erythrocytes coated by the corresponding immunoglobulin).

### Preparation of Sperm Cells and Sperm Cell Lysate

To analyze the differences in cytokine spectra induced by superficial vs. all sperm antigens, we used two types of sperm antigenic stimuli, whole sperm cells and sperm cell lysate. For preparation of those stimuli, we used liquefied semen specimen (30 min, room temperature) from healthy donors obtained by masturbation after four days of total sexual abstinence according to WHO rules, and the sperm cells were separated from seminal plasma using direct swim-up procedure [8]. Briefly, volume of 1 ml was layered gently under 1 ml of modified Baker’s solution (0.44 g of CaCl_2_;0.2 g of KCl; 0.1 g of MgSO_4_·7 H_2_O; 3.4 g of NaCl; 1.1 g of NaHCO_3_; 0.07 g of NaH_2_PO_4_·H_2_O; 0.5 g of Glucose; 8.3 ml of Phenol red; sterile distilled water to 500 ml) and incubated for 1 h at 37°C in a 5% CO_2_ incubator. The swim-up portion, the supernatant with the enriched fraction of motile spermatozoa was collected for further use. Concentration of sperm cells after double swim-up used for PBMC stimulation was 10^6^ per ml of final cultivation volume. Part of the sperm cells was frozen at −20°C for sperm cell lysate preparation.

For sperm cell lysate preparation, the thawed suspension of sperm cells, prepared as described above, was homogenized and centrifuged for 10 min (2100×g, 4°C). The pellet was then washed three times (5 min, 2100×g, 4°C) with phosphate buffered saline (PBS, 8 mM Na_2_HPO_4_, 1.5 mM KH_2_PO_4_, 0.15 M NaCl, 2.7 mM KCl, pH 7.4) containing 150 µl of the protease inhibitor cocktail (Sigma-Aldrich, St Louis, MO, USA, P8340) per 100 ml of PBS. The pellet was solubilized in a lysis buffer containing 0.01 M Na_2_HPO_4_, 0.01 M NaH_2_PO_4_, 0.15 M NaCl, 1% Triton X-100, 1% sodium deoxycholate and protease inhibitor cocktail (Sigma-Aldrich), pH 7.4. Cells were lysed under constant and intensive shaking for 30 min at 4°C. Insoluble material was removed by centrifugation for 10 min at 8500×g at 4°C. Protein concentration was measured by BCA™ Protein Assay Kit (Pierce, Rockford, IL, USA) [7] and equivalent of 10 mg of protein was used for PBMC stimulation.

### Isolation and Cultivation of Peripheral Mononuclear Cells (PBMCs)

PBMCs were isolated from heparinized blood by Ficoll-Hypaque density gradient centrifugation (density 1.077 g/l, Sigma-Aldrich). The cells were washed in sterile PBS and adjusted to the final concentration of 10^7^ PBMCs per ml of serum-free X-VIVO™ 10 medium (Cambrex, East Rutherford, NJ, USA). Total amount of 10^6^ of these cells were then incubated in a humidified incubator for 3 or 5 days at 37°C and 5% CO_2_ in the presence or absence of either sperm cells or sperm cell lysate in total volume of 1 ml. The supernatants from PBMCs cultivated in the absence of any stimulation were used as an inner control. After the cultivation, the supernatants were collected and stored at −70°C until analysis for cytokines.

### Antibody Array

We measured the cytokine spectra in the supernatants after PBMC cultivation using the RayBio® Human Inflammation Antibody Array 3 (RayBiotech, Norcross, GA, USA), which detects 40 cytokines, chemokines and growth factors simultaneously. Array membranes were processed following the manufacturer’s recommendations. The signal intensity was measured on the LAS-1000 luminescence detector (Fujifilm, Tokyo, Japan), and the resulting images were analyzed using AIDA software (Version 3.28, Raytest, Straubenhardt, Germany). To compare the luminescence intensities among the samples, we subtracted the background staining and normalize the data to the positive controls on the same membrane as described previously [29].

### Statistical and Bioinformatic Analysis

For the purpose of statistical analyses by significance analysis of microarrays (SAM), data were transformed to z-scores. The group differences between samples from different groups were analyzed with the SAM method (ver. 3.11; available online at http://www-stat.stanford.edu/~tibs/SAM/) as previously described [30]. The SAM method uses permutations of the repeated measurements to estimate the percentage of proteins identified by chance, the false discovery rate (FDR). Only the molecules with FDR lower than or equal to 10% are shown. The samples were clustered on the basis of similarity in pattern of expression over all samples using unsupervised clustering algorithm with a top down repeated bisection approach in the clustering package CLUTO 2.1.1 (http://glaros.dtc.umn.edu/gkhome/cluto/cluto/download), as described previously [31]. The similarity between the protein productions was calculated using the correlation coefficient.

We compared the cytokine spectra produced by the PBMCs after different stimulation or at different time point (3 vs. 5 day cultivation), using pair-matched two-way analysis of variance (ANOVA). Differences were considered statistically significant at P<0.05. GraphPad Prism statistical software (version 5.03, GraphPad Software, Inc. La Jolla, CA, USA) was used for analyses.
